# Nutrients and Muscle Disease

**DOI:** 10.1155/2015/809830

**Published:** 2015-03-19

**Authors:** Enzo Nisoli, Robert W. Grange, Giuseppe D'Antona

**Affiliations:** ^1^Center for Study and Research on Obesity, Department of Medical Biotechnology and Translational Medicine, University of Milan, Via Vanvitelli 32, 20129 Milan, Italy; ^2^Department of Human Nutrition, Foods and Exercise, College of Agriculture & Life Sciences, Virginia Tech University, Blacksburg, VA 24061, USA; ^3^Department of Molecular Medicine and Laboratory for Motor Activities in Rare Diseases (LUSAMMR), University of Pavia, Via Forlanini 6, 27100 Pavia, Italy

Myopathies, classified as either hereditary or acquired, lead to common, clinically relevant complaints including fatigue, progressive strength loss, myalgias, and cramps. Important progress has been made in the comprehension of the molecular mechanisms underlying muscle diseases but, in the majority of cases, their clinical management is symptom-oriented and includes physical therapy, physical exercise, orthopaedic corrections, pharmacologic interventions, and artificial ventilation.

Previously, there has been a paucity of efficacious available treatments for myopathies, particularly related to the notion that nutrients may exert a variety of positive effects on skeletal muscles. However, more recently there has been an increase in studies that have explored the effects of nutritional interventions. These include changes in macronutrient composition and nutritional supplements to ameliorate the decrements in skeletal muscle structure and function. Indeed, the emerging consensus is that multiple strategies including nutrition and physical exercise may be successful in certain cases. This approach has been mainly supported by the acquired and consolidated knowledge on the ergogenic, trophic, anti-inflammatory, and antioxidant effects of selected nutrients (i.e., amino acids, creatine, carnitine, *ω*3-polyunsaturated fatty acids, vitamin D, and polyphenols) on skeletal muscles that, in part, explains their widespread use within the general population and, more so, by recreational and professional athletes.

Notwithstanding this potential, a number of variables can significantly impact the muscular outcome of nutrients including type, combination, timing of administration, duration of treatment, as well as sex, age of intervention, and genotype of the subjects to be supplemented. Thus, confident conclusions about their use cannot yet be drawn under either normal physiological or pathophysiological conditions. Furthermore, experimental evidence to support both short- and long-term effects and the safety of supplements in the different myopathies are still not well-defined. These limitations have encouraged researchers to investigate the regenerative and anti-inflammatory properties of selected nutrients under simplified experimental settings, such as exercise-induced adaptation to muscle injury due to cycles of repetitive eccentric and concentric contractions. Overall these efforts led to the idea that nutrients may positively impact muscle regeneration by acting at different levels and partly through common mechanisms (e.g., the mTOR pathway) which could be related to degenerative/regenerative responses to damage, including early inflammation, satellite cell activation, proliferation and fusion, neuromuscular junction formation and, finally, maturation of newly formed myofibers.

The special issue includes two original research articles and five comprehensive reviews. What emerges from this special issue is that selected compounds or nutritional approaches could be useful in certain types of muscle pathologies, but, considering the multifaceted etiology of myopathies, there is also the likelihood that each compound or nutritional intervention could be ineffective or even potentially harmful under certain conditions. We hope that this special issue may represent a useful reference and encourage additional studies by investigators to expand knowledge of nutrition as an alternative/supplementary approach to treat muscle diseases.

*Enzo Nisoli* *Robert W. Grange* *Giuseppe D'Antona*

